# Random Subspace Ensemble Learning for Functional Near-Infrared Spectroscopy Brain-Computer Interfaces

**DOI:** 10.3389/fnhum.2020.00236

**Published:** 2020-07-17

**Authors:** Jaeyoung Shin

**Affiliations:** Department of Electronic Engineering, Wonkwang University, Iksan, South Korea

**Keywords:** brain-computer interface, ensemble learning, functional near-infrared spectroscopy, linear discriminant analysis, random subspace, support vector machine

## Abstract

The feasibility of the random subspace ensemble learning method was explored to improve the performance of functional near-infrared spectroscopy-based brain-computer interfaces (fNIRS-BCIs). Feature vectors have been constructed using the temporal characteristics of concentration changes in fNIRS chromophores such as mean, slope, and variance to implement fNIRS-BCIs systems. The mean and slope, which are the most popular features in fNIRS-BCIs, were adopted. Linear support vector machine and linear discriminant analysis were employed, respectively, as a single strong learner and multiple weak learners. All features in every channel and available time window were employed to train the strong learner, and the feature subsets were selected at random to train multiple weak learners. It was determined that random subspace ensemble learning is beneficial to enhance the performance of fNIRS-BCIs.

## Introduction

Ensemble learning has been applied actively in many different machine learning fields (Akram et al., 2015; Li et al., 2016; Ren et al., 2016; Hassan and Bhuiyan, 2017; Sagi and Rokach, 2018; Yaman et al., 2018; Zerrouki et al., 2018). It is defined as a type of machine learning technique that takes advantage of multiple weak (i.e., straightforward but fair performance) learners instead of a single strong (i.e., sophisticated and powerful performance) learner to make high-quality predictions. This implies that the principle of collective intelligence being superior to an elite can be applied in the field of machine learning. Ensemble learning approaches are typically categorized into (i) bootstrap aggregating (bagging), (ii) boosting, and (iii) random subspace (Breiman, 1996; Freund and Schapire, 1997; Ho, 1998). With respect to a structural perspective, which is different from other ensemble approaches mentioned earlier, stacking, namely meta-learning, can be included in ensemble learning.

Bagging algorithms generate multiple (tens or hundreds) bootstrap replicas of an original dataset to train multiple weak learners corresponding to bootstrap replicas. Each bootstrap replica is composed of *N* samples, where *N* is the same as the original dataset size, selected at random with replacement. The algorithm trains weak learners until every weak learner is trained. Boosting algorithms train weak learners sequentially by focusing on the data misclassified by a weak learner. The misclassified data and correctly predicted data increasingly possess higher and lower weights, respectively. The next weak learner is trained on the data with adjusted weights to reduce classification loss. Random subspace algorithm requires less computational cost than others because the method uses random subsets containing *M* features out of *D* features, where *D* is the total number of features. Each weak learner is trained using a random subset of *m* features until every weak learner is trained. Stacking algorithms construct multi-level learners. The gist of this algorithm is that outputs of base learners are used as training data for the higher-level meta-classifier.

In previous studies in the fields of neuroscience and neural engineering, ensemble learning proved its effectiveness in improving classification performance (Cho and Won, 2007; Sun et al., 2007; Kuncheva and Rodriguez, 2010; Kuncheva et al., 2010; Plumpton et al., 2012). Electroencephalography-based brain-computer interface (EEG-BCI) is one of the major topics in the field of neural engineering, and various ensemble methods have been employed successfully to enhance the EEG-BCI performance (Sun et al., 2008; Liyanage et al., 2013). Another case garnering considerable attention is a functional near-infrared spectroscopy-based brain-computer interface (fNIRS-BCI), which already demonstrated its potentials as an alternative to EEG-BCIs owing to its cost-effectiveness, portability, scalability, and convenience (Herrmann et al., 2003; Kubota et al., 2005; Irani et al., 2007; Sitaram et al., 2007; Ye et al., 2009; Zhang et al., 2009; Falk et al., 2011; Power et al., 2011, 2012; Holper et al., 2012; Naseer et al., 2014; Khan and Hong, 2015; Shin et al., 2016; Shin and Im, 2018). Because of their excellent performance and reliability, support vector machine (SVM) and linear discriminant analysis (LDA) are two popular machine learning approaches that implement fNIRS-BCI systems. According to Naseer and Hong (2015), SVM or LDA is utilized in over 65% of fNIRS-BCI studies as a machine learning method. It is noteworthy that only a few studies on ensemble learning for fNIRS-BCI have been conducted to date. Instead, hybrid EEG-fNIRS BCI, which recently proved excellent in BCI performance, plays a leading role in taking advantage of the benefits of ensemble learning for fNIRS-BCIs (Fazli et al., 2012; Shin et al., 2017b, 2018a,c,d, 2019; Von Lühmann et al., 2017; Kwon et al., 2020).

Although different types of fNIRS-BCI related studies have been introduced (Sereshkeh et al., 2019; Ghonchi et al., 2020; Nagasawa et al., 2020; Von Luhmann et al., 2020) Deep recurrent–convolutional neural network for classification of simultaneous EEG–fNIRS signals), surprisingly, little literature has covered the advantages of ensemble learning to improve the performance in terms of classification accuracy, information transfer rate, etc. (Shin and Im, 2020). In this study, the effectiveness of ensemble learning for fNIRS-BCIs is evaluated. For this, the random subspace method takes charge of the core of the ensemble learning algorithm used in this study. Classification accuracies that are yielded by a single strong learner and an ensemble of multiple weak learners are provided as proof of validation results.

## Methods

### Open-Access Dataset

**Figure 1:**
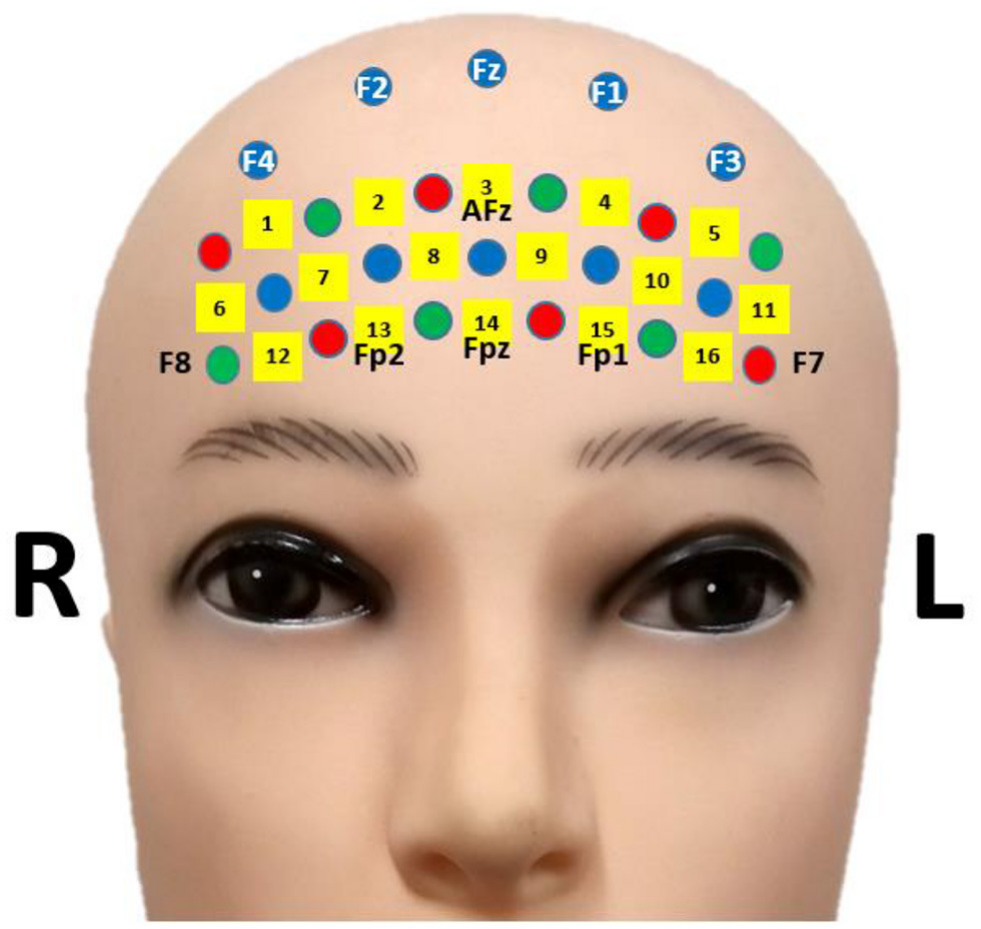
fNIRS channel location. The figure is adapted from Shin et al. (2018c) under CC-BY license.

An open-access fNIRS-BCI dataset was used in subsequent data analyses to secure the reproducibility and accuracy of results. The fNIRS-BCI dataset can be downloaded using the URL (Shin et al., 2019). The dataset included a 16-channel fNIRS data of 18 participants [10 males and eight females, 23.8 ± 2.5 years (mean ± standard deviation)]. Optical intensity changes (Δ[*OD*]) were collected by a multi-channel fNIRS device (LIGHTNIRS; Shimadzu Corp.; Kyoto, Japan) utilizing three wavelengths (780, 805, and 830 nm) at a sampling rate of 13.3 Hz. The location of fNIRS channels is presented in Figure 1 (Shin et al., 2018c). Participants performed: (i) mental arithmetic task (repetitive subtractions of a one-digit number from a three-digit number) and (ii) stayed in an idle state during the task period of 10 s and relaxed during the following rest period (24~26 s). Items (i) and (ii) were repeated 30 times. Sixty-trial fNIRS data were collected from each participant after data recording. The details can be found in Shin et al. (2018c).

### Preprocessing

The collected Δ[*OD*] were converted to two types of fNIRS chromophore data (i.e., concentration changes in oxygenated hemoglobin Δ[*HbO*] and reduced hemoglobin Δ[*HbR*]) using the following formula which is given by Matcher et al. (1995):

$$\begin{array}{l} \left(\begin{array}{c} \Delta \left[HbR\right]\\ \Delta [HbO]\; \end{array}\right)=\left(\begin{array}{ccc} 1.8545 & -0.2394 & -1.0947\\ -1.4887 & 0.5970 & 1.4847 \end{array}\right)\;\\ \;\cdot \;\left(\begin{array}{c} \Delta {\left[OD\right]}_{780}\\ \Delta {\left[OD\right]}_{805}\\ \Delta {\left[OD\right]}_{830} \end{array}\right)\;\cdot \;(\text{unit}:\text{ mM}\cdot \text{cm}) \end{array}$$

where the subscript denotes a wavelength.

The converted data were band-pass filtered using a sixth-order Butterworth zero-phase filter with a pass-band of 0.01–0.09 Hz (Shin et al., 2017a, 2018b,c). The pass-band was selected to eliminate unwanted physiological noises and DC offsets (Zhang et al., 2007). Any motion artifact removal method was not applied because the fNIRS data were collected at the stationary state. The filtered data were segmented into epochs ranging from −1 to 15 s relative to task onset (0 s). Afterward, the segmented data were subjected to a baseline correction to subtract each channel offset in the reference interval (−1 to 0 s).

## Classification

### Features

A variety of features have been considered in fNIRS-BCI studies such as mean, slope, variance, etc. Among them, mean (average amplitude of fNIRS data; AVG) and slope (average rate of amplitude change of fNIRS data; SLP) were the most relevant features in previous fNIRS studies (Bhutta et al., 2015; Hong and Santosa, 2016; Hong et al., 2017; Shin et al., 2018c); therefore, four types of time windows were employed to extract AVG and SLP of the segmented Δ[HbO] and Δ[HbR] data ranging from 0 to 15 s. Each type of window subdivided the interval into 1, 3, 5, and 15.

- TYPE 1: single time window with a length of 15 s.
- TYPE 2: three time windows with a length of 5 s: [0 5], [5 10], and [10 15] s.
- TYPE 3: five time windows with a length of 3 s: [0 3], [3 6], …, [12 15] s.
- TYPE 4: fifteen time windows with a length of 1 s: [0 1], [1 2], …, [14 15] s.

Feature vectors were constructed using both AVG and SLP as well as a single feature, either AVG or SLP. The dimensionality of feature vectors (i.e., the number of features, *D*) for each of the trials is given as:

(1) $$\begin{array}{l} D=n_{type}\;\times n_{chrm}\times n_{ch}\times n_{win} \end{array}$$

where *n*_*type*_, *n*_*chrm*_, *n*_*ch*_, and *n*_*win*_ are the number of feature types (1 or 2), chromophores (2), channels (16), and time windows (1, 3, 5, or 15 for TYPE 1, 2, 3, or 4), respectively.

### Strong Learner

As mentioned in the Introduction section, the two most popular machine learning algorithms (SVM and LDA) were employed. Feature vector standardization was applied to linear SVM to improve prediction performance while other miscellaneous hyperparameters were default values. However, the feature vector standardization was not applied to LDA because, as understood, the application of standardization does not significantly affect LDA prediction performance. The number of *D* features was used to train strong learners.

### Random Subspace

LDA was chosen as a type of weak learner. Random subsets of *M* features out of *D* features were used to train *N* weak learners, where *M* = $\left\{m\;|\;m=\left[\sqrt{D}+0.5\right]\pm 2,\pm 4,m\;\in \mathbb{N},\;D\;\in \mathbb{N}\right\}$, [·] operator denotes the integer part of a number (e.g., *M* = {3, 5, 7, 9, 11}, *D* = 50, $\left[\sqrt{D}+0.5\right]=7$), and *N* = {*n* | 1 ≤ *n* ≤ 100, *n* ∈ ℕ}. The basic rule of random subspace ensemble follows the steps below:

- choose *N* subsets containing *M* features selected at random from *D* features.
- train *N* weak learners using each random subset.
- make a prediction by majority vote.

### Cross-Validation

In the case of the strong learner, a 10 × 10-fold cross-validation was performed to estimate the generalized classification performance. A 60-trial training set for a single participant was divided into 10-folds. A training dataset and a test set were composed of 9-folds and the rest data, respectively. The strong learner was trained using the training set, and the classification performance was validated using the test set, which included unseen data during the training process. The validation was repeated until every fold was used at least once to estimate the classification performance. In the case of ensemble learning, while steadily increasing the number of weak learners up to *N*, ten repetitions of 10-fold cross-validation were performed using the training and test sets partitioned in the same way, as employed in the process of strong learning validation.

### Test Statistic

Prediction performances of random subspace ensembles and strong learners were tested by repeated cross-validation tests. The assessment was conducted as follows:

- compute differences between classification losses for *k*^*th*^fold of *r*^*th*^ repetition of a random subspace ensemble and a strong learner:
  (2) $$\begin{array}{l} d_{r,k}=\;err_{ens\left(r,k\right)}-\;err_{svm\left(r,k\right)} \end{array}$$
- compute the average differences across *K* folds:
  (3) $$\begin{array}{l} E\left(d_{r}\right)=\frac{1}{K}\overset{K}{\underset{k=1}{\sum }}d_{r,k} \end{array}$$
- compute average differences across *R* repetitions:
  (4) $$\begin{array}{l} E\left(d\right)=\frac{1}{R}\overset{R}{\underset{r=1}{\sum }}E\left(d_{r}\right) \end{array}$$
- compute variances of the differences:
  (5) $$\begin{array}{l} \sigma_{r}^{2}=\frac{1}{K}\sum_{k=1}^{K}{\left(d_{r,k}-E(d_{r})\right)}^{2} \end{array}$$
- compute average variances across *R* repetitions:
  (6) $$\begin{array}{l} E\left(\sigma^{2}\right)=\frac{1}{R}\overset{R}{\underset{r=1}{\sum }}\sigma_{r}^{2} \end{array}$$
- compute overall variances of the differences:
  (7) $$\begin{array}{l} S^{2}=\frac{1}{RK}\overset{R}{\underset{r=1}{\sum }}\overset{K}{\underset{k=1}{\sum }}{\left(d_{r,k}-E\left(d\right)\right)}^{2} \end{array}$$

A test statistic (*t*) for comparing classification losses of both random subspace ensemble and the strong learner is given by:

(8) $$\begin{array}{l} t\;=\frac{E\left(d\right)}{\sqrt{S^{2}/\left(df+1\right)}} \end{array}$$

where *df* is a degree of freedom and was assigned 10 in this study (Bouckaert and Frank, 2004; Wang et al., 2017).

### Statistical Test

Parametric statistical methods, such as analysis of variance (ANOVA) and *t*-test, were adopted because the Anderson-Darling test for classification accuracies returned a test decision indicating that the classification accuracies were from a population with a normal distribution.

## Results

### Strong Learners

**Figure 2:**
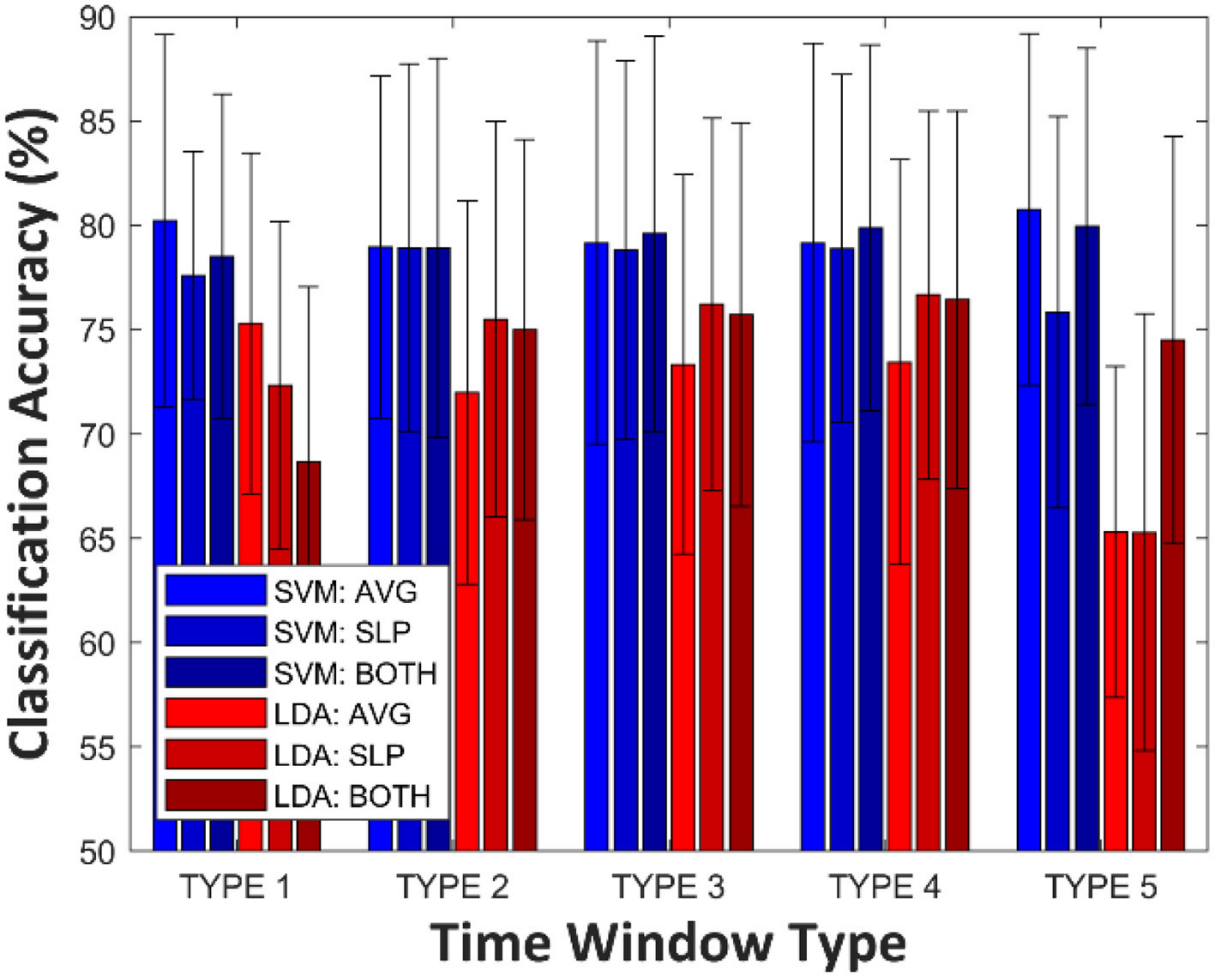
Grand averages (over all participants) of LDA and SVM classification accuracies according to time window type. The error-bar indicates standard deviation.

Figure 2 shows grand averages (over all participants) of LDA and SVM classification accuracies according to the time window type for constructing feature vectors. Variance analysis (ANOVA) was employed to test whether a significant difference existed among single-trial classification accuracies corresponding to each of the feature vectors. The significant variability of SVM classification accuracy by different time window and feature types (One-Way ANOVA, *p* = 0.991) was not observed; in other words, the SVM prediction power was not significantly influenced by the number of dimensions (i.e., how many features) and type of features that were considered, at least in this study. Instead, for SVM: AVG at TYPE 5, the best grand average classification accuracy yielded a result of 80.8 ± 8.4% (mean ± std), although this is not statistically significant, considering the highest-dimensional feature vectors. However, statistically, different prediction performances were observed among LDA classifiers (One-Way ANOVA, *p* < 0.001). In the case of TYPE 5, LDA classification accuracies, estimated by a single type of feature (AVG: 65.3 ± 7.9%, SLP: 65.3 ± 10.5%), decreased steeply below the effective binary BCI threshold [commonly, 70.0% (Dickhaus et al., 2009; Allison and Neuper, 2010; Vidaurre and Blankertz, 2010)].

### Random Subspace Ensemble

**Figure 3:**
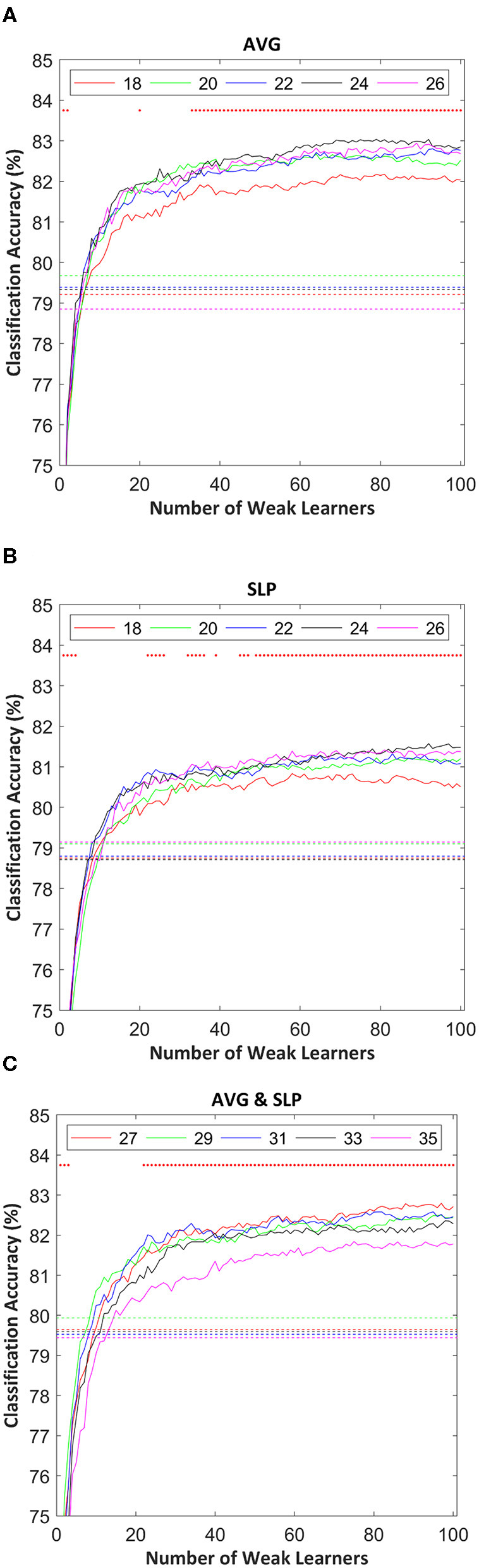
Random subspace ensemble classification accuracies estimated using: **(A)** AVG, **(B)** SLP, and **(C)** AVG and SLP features as a function of the number of weak learners. Dotted lines indicate SVM (strong learner) classification accuracy, estimated using the same 10-fold cross-validation partition used to validate the classification performance of the random subspace ensemble classifier. The random subset size for each weak learner is shown in the legend. Red dots in the upper part of each subfigure indicate the significance of differences in the classification accuracy between ensembles and strong leaners (*t*-test with false discovery rate corrected-*p* < 0.05).

Classification accuracies of random subspace ensemble classifiers varied as a function of the number of weak learners as shown in Figure 3. Typically, classification accuracies were drastically improved until the 20 weak classifiers were involved in the ensemble. Afterward, it was confirmed that the classification accuracy gradually increased as the number of weak learners increased. The classification enhancement rate distinctly became lower in the regions where the ensemble included around 80 or more weak learners.

Overall, ensembles with 10+ weak learners outperformed a single strong learner, and this improvement was significantly observed in the areas where the number of weak learners was > 20 (paired *t*-test with false discovery rate corrected-*p* < 0.05). The highest ensemble classification accuracy was often yielded where the subset size was relatively larger; however, its consistency was not observed. Table 1 shows the highest individual random subspace ensemble classification accuracies, estimated using the fittest subset size. Using either AVG or SLP feature, a classification accuracy of 84.5 ± 6.2 or 83.0 ± 6.4%, respectively, was obtained on average. By using both features simultaneously, 84.4 ± 6.5% classification accuracy was obtained on average. The difference in classification accuracy among these three cases was not significant (One-Way ANOVA, *p* = 0.746). It was rarely displayed that *N* was <20 (3 out of 54 cases), while diverse *M*-values were selected depending on each participant to obtain the highest classification accuracy for each participant.

**Table 1:** The best classification accuracy of the ensemble that contains *N* weak learners, which were trained using random feature subsets of the fittest size (*M*).

	**AVG**			**SLP**			**AVG and SLP**		
**Participant**	**Acc**.	***N***	***M***	**Acc**.	***N***	***M***	**Acc**.	***N***	***M***
1	0.852	87	18	0.880	92	26	0.847	97	27
2	0.855	43	26	0.815	33	26	0.838	98	33
3	0.823	27	22	0.808	100	26	0.828	81	27
4	0.960	84	26	0.923	22	22	0.960	87	33
5	0.798	71	18	0.795	32	20	0.778	33	27
6	0.892	73	26	0.867	33	22	0.902	45	33
7	0.740	46	20	0.668	33	24	0.682	32	31
8	0.815	92	26	0.845	38	18	0.830	94	27
9	0.812	95	26	0.767	66	26	0.798	89	27
10	0.758	12	18	0.812	9	22	0.820	28	33
11	0.837	83	24	0.883	65	22	0.873	82	35
12	0.807	34	26	0.775	23	22	0.812	32	31
13	0.777	79	24	0.753	24	20	0.778	84	33
14	0.933	71	24	0.872	63	26	0.935	86	27
15	0.873	49	22	0.858	92	24	0.868	58	27
16	0.935	93	18	0.920	42	18	0.923	14	27
17	0.907	71	20	0.857	59	20	0.868	96	29
18	0.837	84	18	0.848	97	26	0.848	42	33
**Mean**	**0.845**	–	–	**0.830**	–	–	**0.844**	–	–
**Std**	**0.062**	–	–	**0.064**	–	–	**0.065**	–	–

*Bold indicates Standard deviation of individual classification accuracies (Std) and Average classification accuracy across participants (Mean)*.

## Discussion

### Dimensionality

For classification problems, it is generally acknowledged that as the dimensionality of the feature vector is much larger than the training set size, prediction performance can be adversely affected. This is called the “curse of dimensionality.” In areas where only AVG or SLP was used in this study, the ratio of the dimensionality of a feature vector to the training set size was 8.9 approximately (dimensionality: 32 × 15, training set size: 56 based on 10-fold cross-validation partition). The SVM prediction performance did not sensitively suffer from the “curse of dimensionality.” In contrast, a high-dimensional feature vector significantly influenced LDA prediction in consideration of poor classification accuracies in the TYPE 5 case. Regularization can be a viable option to mitigate the adverse effects of high-dimensional feature vectors. If the dimensionality of feature vectors exceeds a training set size, the parameter estimations required for LDA can be highly unstable (e.g., poor covariance matrix estimator; Friedman, 1989). By employing the regularization factor, one can improve the parameter estimations to be more plausible. In such cases, it is essential to select the regularization factor appropriately. It is noted that this approach is often iterative and requires heavy computation. SVM classification performance, at least in this study, was not sensitive to the negative effect of high-dimensional feature vectors. Therefore, if SVM is chosen, regularization, feature selection, and dimension reduction methods need not be applied to alleviate the “curse of dimensionality.”

### Within-Fold Comparison

**Figure 4:**
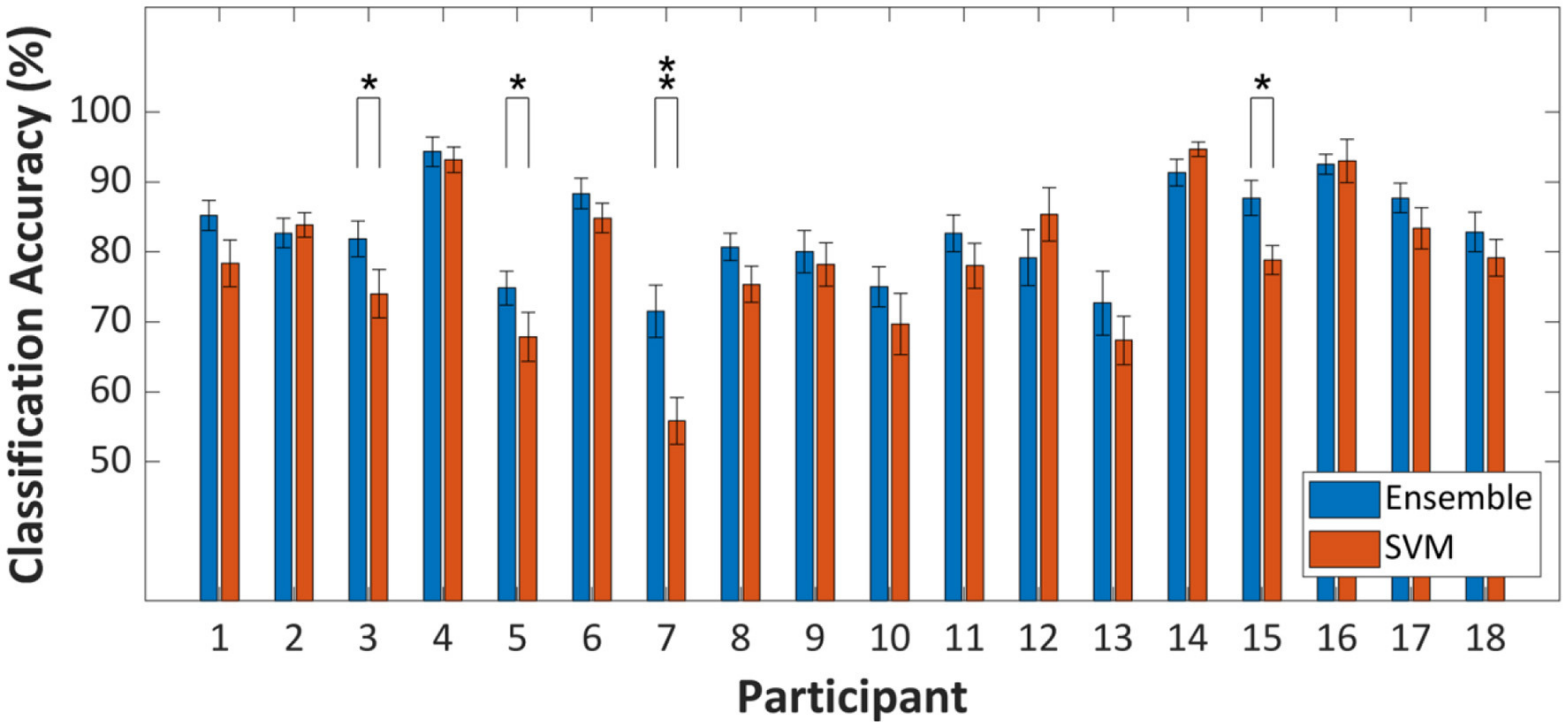
Within-fold differences of prediction performance between random subspace ensemble and strong learner (SVM) assessed by ten repetitions of a 10-fold cross-validation-based *t*-test. ^*^p < 0.05, ^**^p < 0.01. Error-bars represent the standard deviation.

In terms of the classification accuracy improvement at the group level (i.e., grand average classification accuracy), a strong statistical proof, which infers that ensemble learning is more beneficial than a strong classifier, was provided. On the other hand, fold-wise comparisons of classification accuracies of SVM and random subspace ensemble to investigate classification accuracy improvement at the individual level results in somewhat different statistical results. The degree of improvement is not revealed as statistically significant for most individual cases. Figure 4 shows the within-fold differences in classification accuracy between ensemble and strong learner. As shown in Figure 3, while significant within-group differences in classification accuracy were observed, 14 out of 18 individual cases in Figure 4 showed insignificant within-fold differences in classification accuracy. These individual cases were presented (repeated cross-validation *t*-test, *p* > 0.05) because the test statistic was more likely to yield a conservative test decision (i.e., strict to false-positive cases). The null hypothesis is rejected only if the within-fold differences in classification accuracy are consistent. This type of test statistic has not typically been employed for comparison of classifier performance in existing fNIRS-BCI studies. The test statistic might suit cases where the dataset size is too small.

### Features

The main advantage of the random subspace method is to randomly select feature subsets, resulting in low-correlated multiple weak learners. It is well known that prediction performance is usually enhanced where predictions are determined with the help of low-correlated multiple weak learners. However, if most of the features are highly correlated, randomly selected features contained in a feature subset are also likely to be highly correlated. In many fNIRS-BCI studies, features extracted in the time domain such as mean, maximum, variance, and slope are correlated (Hwang et al., 2014). Moreover, because NIRS signals are slowly-varying continuous signals, features extracted from different time windows are likely to be correlated as well (Cui et al., 2010a,b). As seen in Table 2, random subspace ensemble learners trained using features extracted in different numbers and sizes of time windows show very similar prediction performance to one another (One-Way ANOVA, *p* = 0.995). Features extracted in different ways can be added by gaining higher prediction performance, rather than by typical algebraic methods in the time domain.

**Table 2:** Comparison of random subspace ensemble classification accuracies according to type of time window (AVG, *N* = 100, *M* = 22).

	**Type 2**	**Type 3**	**Type 4**
**Mean**	0.827	0.827	0.829
**Std**	0.072	0.065	0.067
One-Way ANOVA *p*		0.995	

### Future Prospects

The ensemble classifier has better classification accuracy; however, the ensemble method that requires far more computing power will not be absolutely the first option in any case. It is anticipated that a conventional single strong learner, such as SVM, LDA, etc., could be a more proper option under the circumstance like a real-time system with low computational resources. However, where the computing power is sufficient, ensemble learning methods have the immanent potential to replace the existing single strong learner. Even though deep learning has been receiving much attention recently, it is very difficult to collect enough data for fNIRS deep learning to improve classification performance, and thereby it lacks practicality. Recent findings showed the feasibility of deep learning on fNIRS-studies. However, their finding is not enough to be accepted generally and to be proven rigorously. Hence, ensemble learning as an alternative to deep learning is also likely to attract attention to improve fNIRS-BCIs.

## Conclusion

In this study, the enhanced prediction performance of random subspace ensemble learning was validated to investigate the feasibility of ensemble learning based on the random subspace method. The two most popular types of temporal fNIRS signal features called AVG and SLP were employed to estimate the classification performance of both single strong learners and ensembles based on the random subspace method. Ensembles containing more than 20 LDA weak learners outperformed SVM strong learners significantly. The use of different single temporal features such as AVG and SLP did not make a significant difference in the prediction performance of ensembles. This study is expected to be helpful in the comprehension and use of ensemble learning in future fNIRS-BCI studies.

## Data Availability Statement

The datasets analyzed in this study can be found at the following source: https://doi.org/10.6084/m9.figshare.9198932.v1.

## Author Contributions

JS managed all works for this study. The author confirms being the sole contributor of this work and has approved it for publication.

## Conflict of Interest

The author declares that the research was conducted in the absence of any commercial or financial relationships that could be construed as a potential conflict of interest.
