# Supplementary material for: Random Subspace Ensemble Learning for Functional Near-Infrared Spectroscopy Brain-Computer Interfaces
Source: Front Hum Neurosci. 2020 Jul 17;14:236. doi: 10.3389/fnhum.2020.00236 (PMC7379868; doi:10.3389/fnhum.2020.00236)
Supplement: Supplementary file 1 [file Table_1.DOCX]

**Random Subspace Ensemble Learning for functional Near-Infrared Spectroscopy Brain-Computer Interfaces**

Jaeyoung Shin

Department of Electronic Engineering, Wonkwang University, Iksan, 54538, Korea

## SUPPLEMENTARY MATERIAL


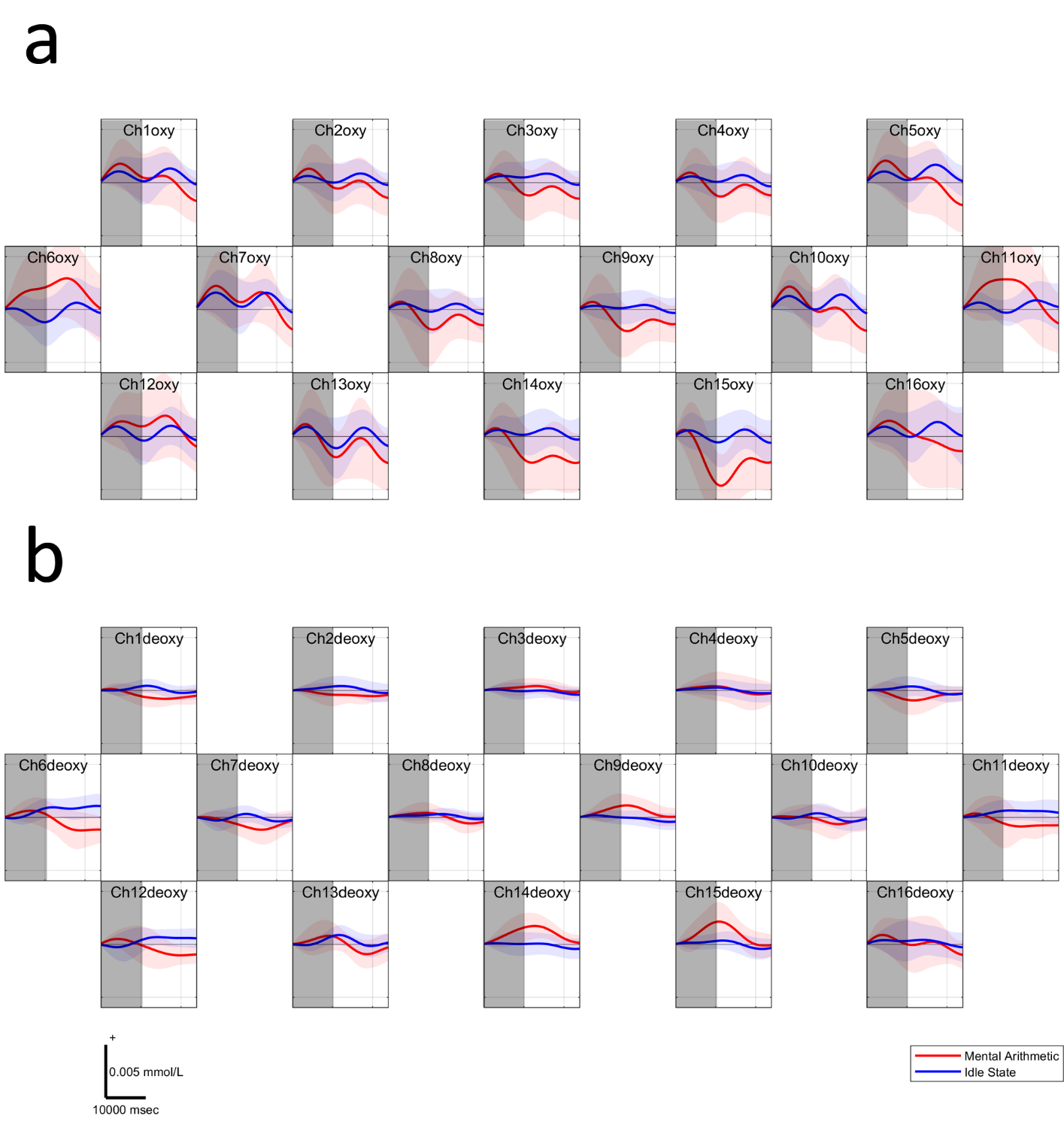


FIGURE S1. Concentration changes of (a) HbO and (b) HbR. Gray shades represent the task period of 10 s. Shaded areas around solid lines indicate the standard deviation.
